# Night Blindness and Day Blindness

**Published:** 1908-10-10

**Authors:** 


### OPHTHALMOLOGY.

### NIGHT BLINDNESS AND DAY BLINDNESS.

Two statements occasionally made by patients are often both put down as being evidence of functional neurosis; but in both there is frequently a very solid foundation for the statement, and one also which can be seen with the ophthalmoscope. The one is that they cannot see at night, the other the converse of this, that they can see best at night or in the dusk, but cannot see so well in daylight.

Patients afflicted with night blindness, or hemeralopia, state that in daylight they can see perfectly well, but that directly it gets dusk they run into things and frequently fall over the kerbstone in the streets. This is no mere neurosis, but a very serious reality, and one very difficult to relieve or cure. Such patients make a point of always being home by dusk or of getting, at least, into a familiar neighbourhood. The causes of this affliction are, primarily, any condition which diminishes the functional activity of the retina or of the light-perceiving elements in the retina; secondarily, opacities in the cornea, lens, and media. The degree of dilatation of the pupil may greatly influence the latter, but in no way affects the former conditions. The most severe form of night-blindness is met with in retinitis pigmentosa. This disease appears to be a slow degeneration or atrophy rather than an actual inflammation of the retina, characterised by deposition of black pigment in the inner layers (next to the vitreous) chiefly along the blood vessels, while the normally pigmented hexagonal epithelial cells of the retina become slowly decolorised and atrophied, and thus the choroidal vessels come more and more into evidence. The rods and cones and the ganglion cells atrophy, and the nerve-fibre layer is probably involved consecutively, while the supporting tissue of the retina becomes hypertrophied. The essential condition is an atrophy of the light-perceiving elements—the rods and cones—the migration or transference of the pigment being quite a secondary consideration.

In a degeneration of the retina closely allied to retinitis pigmentosa the retina becomes uniformly studded with small white dots (retinitis punctata albescens), night-blindness similarly occurring. The same symptoms may be met with in non-pigmented degeneration. Retinitis in any form may give rise to night-blindness during the active inflammatory stage. After the attack the retina may never completely regain its normal activity; hence, when illumination is lessened—as in the dusk—the retina is not so actively stimulated, and cannot recognise the lesser stimulus. Retino-choroiditis also gives rise to night-blindness, especially when extensive and of specific origin.

A less severe form of night-blindness is met with in patients who are suffering from great debility and general bodily mal-nutrition. The vision in daylight and by bright artificial light is perfectly good, but directly the illumination is lessened it diminishes with extraordinary rapidity. In dull light, when the normal sighted can distinguish objects (not only see them but distinguish what they are) the night-blind cannot see at all to such an extent that they will frequently fall over an object standing in their path. Clear bright images formed on the retina stimulate it to normal activity, but dull or less brilliant images apparently do not stimulate it at all. In these cases, if the light sense—that is the appreciation by the retina of differences of illumination—be tested it will be found that it is subnormal—very bright illumination is alone recognisable, and anything less is practically darkness. This condition can often be relieved in time with the aid of tonics, suitable feeding, and improved hygienic surroundings.

Temporary night-blindness occurs in some cases of jaundice, chronic alcohol poisoning, malaria, and the last few months of pregnacy; also in true scurvy and in scurvy-rickets. Opacities in the periphery of the lens and cornea sometimes give rise to minor degrees of night-blindness. In daylight or under brilliant illumination the pupil is contracted and thus the rays enter through clear media and good vision is the result. In darkness, however, the pupils are much more dilated, and as the peripheral rays enter through opacities they are broken up and cannot carry with them a clear image.

Patients with high degrees of uncorrected hypermetropia and myopia always see much worse at night, for the simple reason that the only images which can be formed on their retinæ are blurred ones of diffusion circles, and diminished illumination deprives them of their only means of compensation. Opacities in the vitreous cause defective vision even in bright daylight by diffusing or deflecting the rays of light after they have entered the pupil, so that only weak stimulation of the retina takes place, and this is still more the case at night.

The converse of night-blindness is nyctalopia. This condition is also brought about by affections of the light-perceiving elements, and by opacities in the cornea, lens, and media. In night-blindness the peripheral portions of the retina are the first affected, and patients suffer by the diminution of the field of vision in not being able to see anything beyond what they directly look at; but in nyctalopia it is central vision which is dulled, and the patient depends upon his peripheral vision for orientation. As this may be quite good he accordingly sees better in the dusk. Tobacco amblyopia gives rise to this symptom much more commonly than does any other cause. So does any affection of the optic nerve which produces a central scotoma, such as retro-bulbar neuritis. The opacities in cornea, lens, and media which give rise to nyctalopia are those situated centrally in the direct line of sight; in daylight when the pupil is contracted vision can only take place through the opacity, and for this reason is defective. At night, however, when the pupil is much larger, vision can take place through the clear media round the central opacity, and is accordingly much better. This condition is met with in patients who have a small central corneal opacity, or commencing nuclear cataract. It must not be overlooked that hemeralopia and nyctalopia are not diseases in themselves, but merely symptoms.

